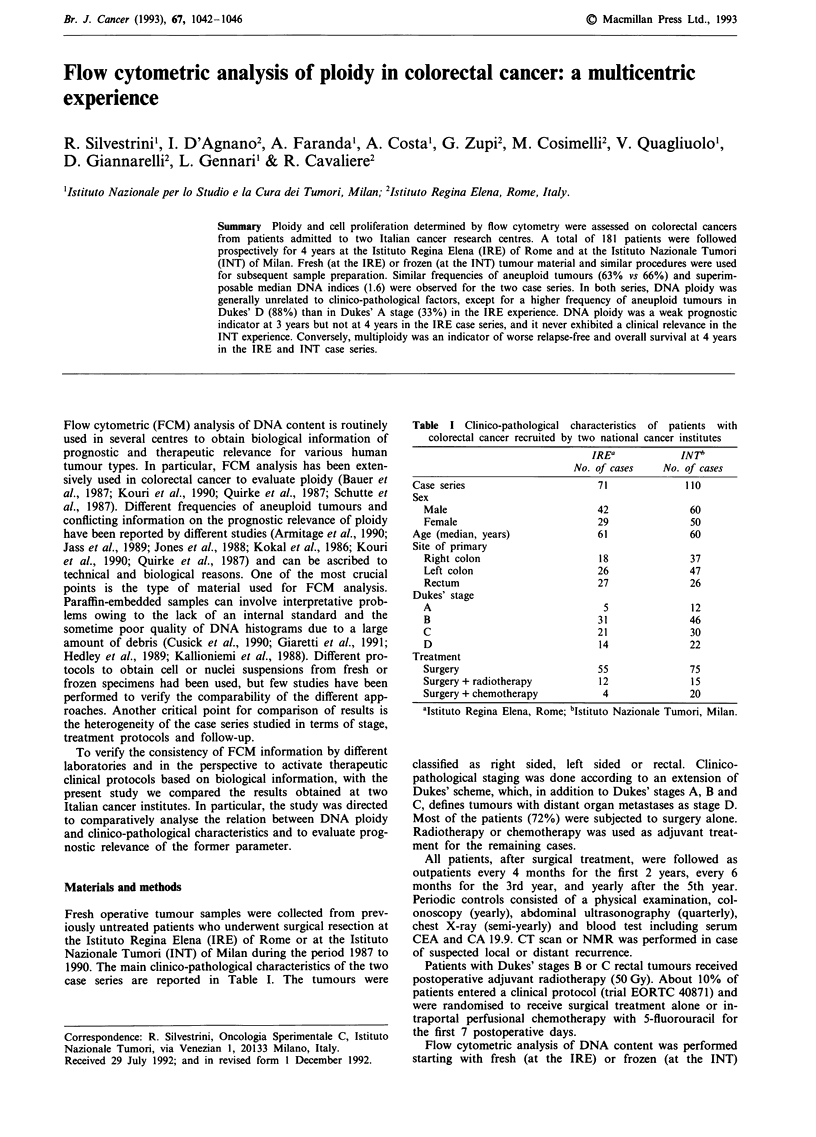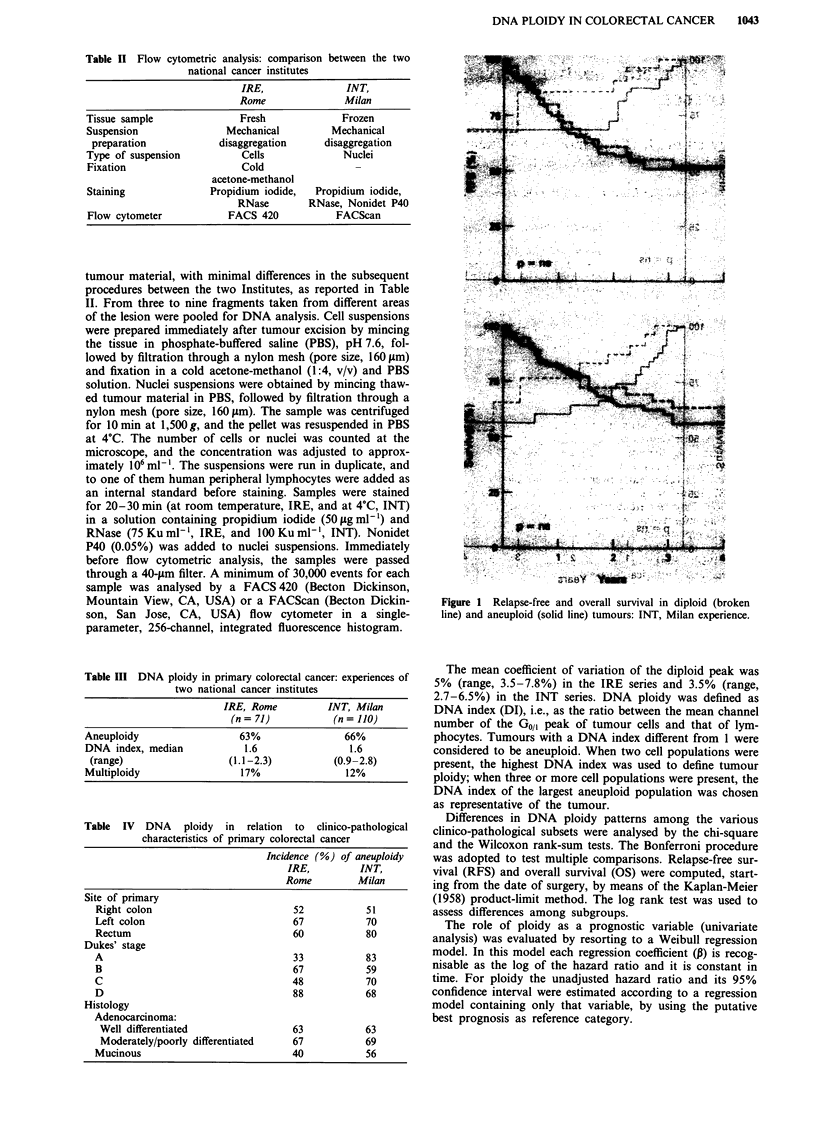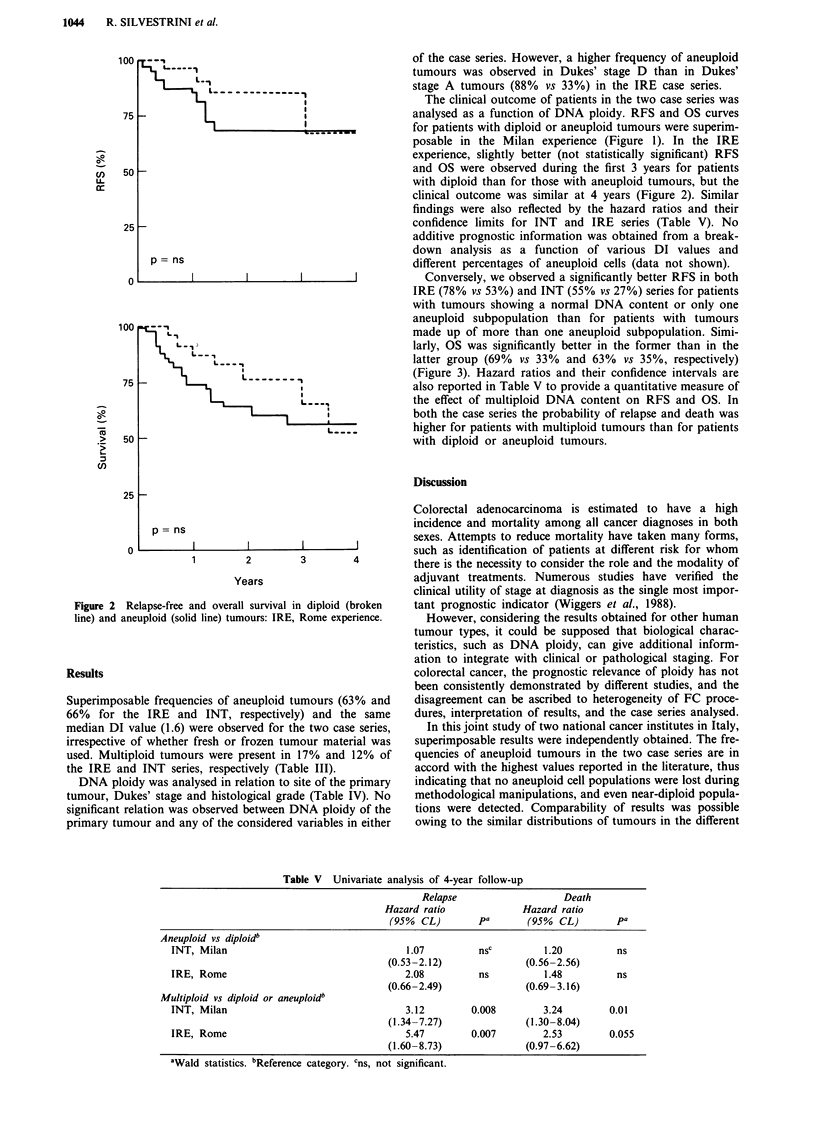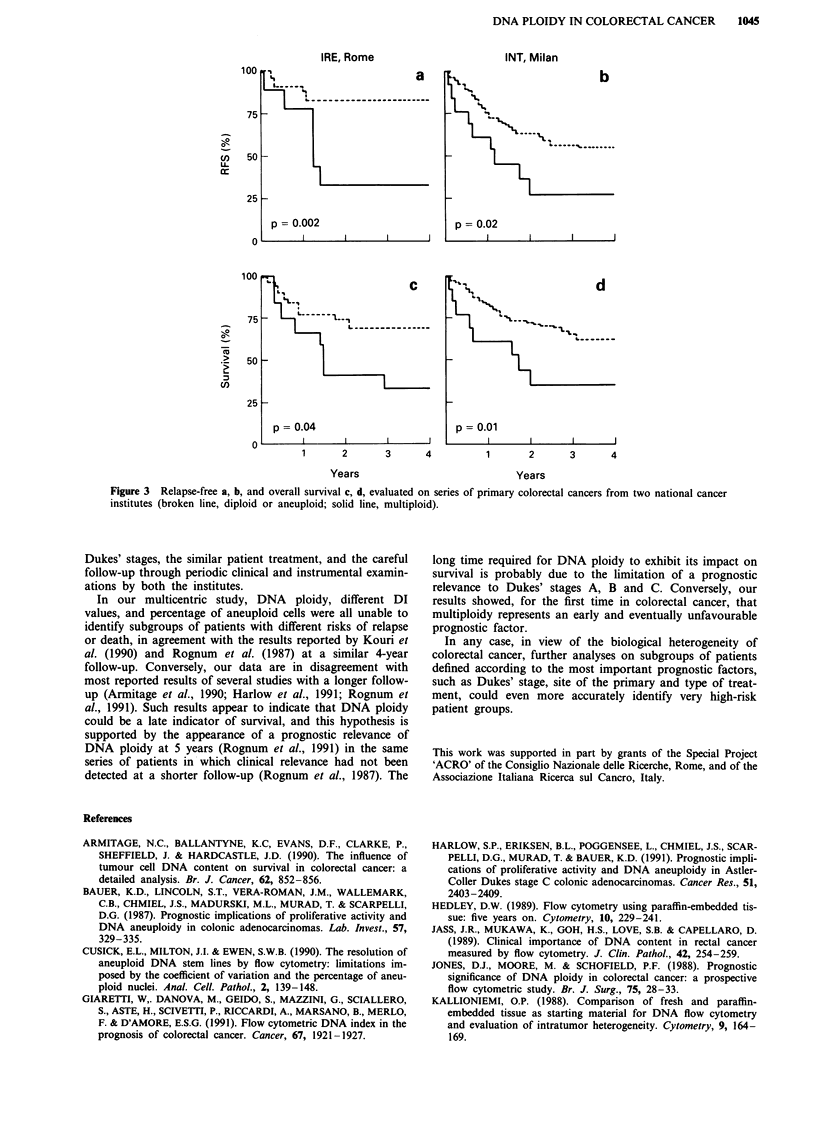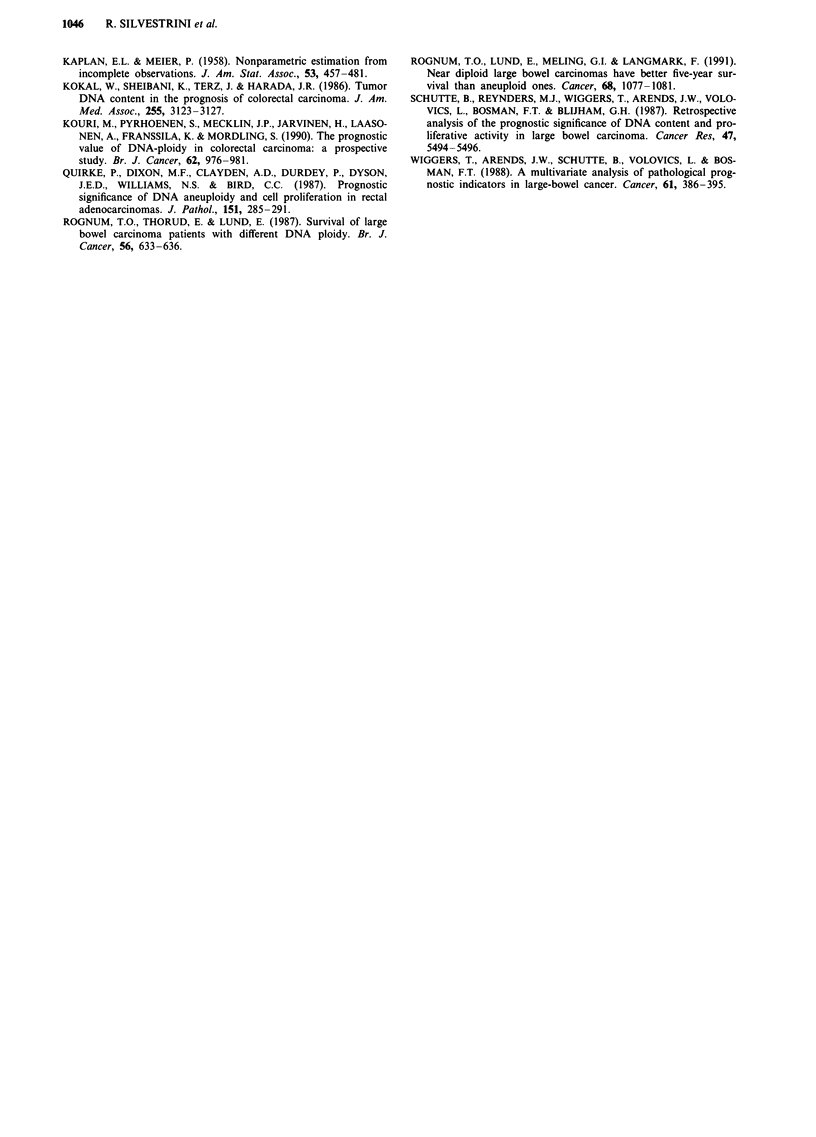# Flow cytometric analysis of ploidy in colorectal cancer: a multicentric experience.

**DOI:** 10.1038/bjc.1993.191

**Published:** 1993-05

**Authors:** R. Silvestrini, I. D'Agnano, A. Faranda, A. Costa, G. Zupi, M. Cosimelli, V. Quagliuolo, D. Giannarelli, L. Gennari, R. Cavaliere

**Affiliations:** Istituto Nazionale per lo Studio e la Cura dei Tumori, Milan, Italy.

## Abstract

Ploidy and cell proliferation determined by flow cytometry were assessed on colorectal cancers from patients admitted to two Italian cancer research centres. A total of 181 patients were followed prospectively for 4 years at the Istituto Regina Elena (IRE) of Rome and at the Istituto Nazionale Tumori (INT) of Milan. Fresh (at the IRE) or frozen (at the INT) tumour material and similar procedures were used for subsequent sample preparation. Similar frequencies of aneuploid tumours (63% vs 66%) and superimposable median DNA indices (1.6) were observed for the two case series. In both series, DNA ploidy was generally unrelated to clinico-pathological factors, except for a higher frequency of aneuploid tumours in Dukes' D (88%) than in Dukes' A stage (33%) in the IRE experience. DNA ploidy was a weak prognostic indicator at 3 years but not at 4 years in the IRE case series, and it never exhibited a clinical relevance in the INT experience. Conversely, multiploidy was an indicator of worse relapse-free and overall survival at 4 years in the IRE and INT case series.


Br. J. Cancer (1993), 67, 1042–1046 © Macmillan Press Ltd., 1993

### Flow cytometric analysis of ploidy in colorectal cancer: a multicentric experience

R. Silvestrini[1], I. D'Agnano[2], A. Faranda[1], A. Costa[1], G. Zupi[2], M. Cosimelli[2], V. Quagliuolo[1], D. Giannarelli[2], L. Gennari[1] & R. Cavaliere[2]

[1]*Istituto Nazionale per lo Studio e la Cura dei Tumori, Milan;* [2]*Istituto Regina Elena, Rome, Italy.*

**Summary** Ploidy and cell proliferation determined by flow cytometry were assessed on colorectal cancers from patients admitted to two Italian cancer research centres. A total of 181 patients were followed prospectively for 4 years at the Istituto Regina Elena (IRE) of Rome and at the Istituto Nazionale Tumori (INT) of Milan. Fresh (at the IRE) or frozen (at the INT) tumour material and similar procedures were used for subsequent sample preparation. Similar frequencies of aneuploid tumours (63% *vs* 66%) and superimposable median DNA indices (1.6) were observed for the two case series. In both series, DNA ploidy was generally unrelated to clinico-pathological factors, except for a higher frequency of aneuploid tumours in Dukes' D (88%) than in Dukes' A stage (33%) in the IRE experience. DNA ploidy was a weak prognostic indicator at 3 years but not at 4 years in the IRE case series, and it never exhibited a clinical relevance in the INT experience. Conversely, multiploidy was an indicator of worse relapse-free and overall survival at 4 years in the IRE and INT case series.

Flow cytometric (FCM) analysis of DNA content is routinely used in several centres to obtain biological information of prognostic and therapeutic relevance for various human tumour types. In particular, FCM analysis has been extensively used in colorectal cancer to evaluate ploidy (Bauer *et al.*, 1987; Kouri *et al.*, 1990; Quirke *et al.*, 1987; Schutte *et al.*, 1987). Different frequencies of aneuploid tumours and conflicting information on the prognostic relevance of ploidy have been reported by different studies (Armitage *et al.*, 1990; Jass *et al.*, 1989; Jones *et al.*, 1988; Kokal *et al.*, 1986; Kouri *et al.*, 1990; Quirke *et al.*, 1987) and can be ascribed to technical and biological reasons. One of the most crucial points is the type of material used for FCM analysis. Paraffin-embedded samples can involve interpretative problems owing to the lack of an internal standard and the sometime poor quality of DNA histograms due to a large amount of debris (Cusick *et al.*, 1990; Giaretti *et al.*, 1991; Hedley *et al.*, 1989; Kallioniemi *et al.*, 1988). Different protocols to obtain cell or nuclei suspensions from fresh or frozen specimens had been used, but few studies have been performed to verify the comparability of the different approaches. Another critical point for comparison of results is the heterogeneity of the case series studied in terms of stage, treatment protocols and follow-up.

To verify the consistency of FCM information by different laboratories and in the perspective to activate therapeutic clinical protocols based on biological information, with the present study we compared the results obtained at two Italian cancer institutes. In particular, the study was directed to comparatively analyse the relation between DNA ploidy and clinico-pathological characteristics and to evaluate prognostic relevance of the former parameter.

### Materials and methods

Fresh operative tumour samples were collected from previously untreated patients who underwent surgical resection at the Istituto Regina Elena (IRE) of Rome or at the Istituto Nazionale Tumori (INT) of Milan during the period 1987 to 1990. The main clinico-pathological characteristics of the two case series are reported in Table I. The tumours were classified as right sided, left sided or rectal. Clinico-pathological staging was done according to an extension of Dukes' scheme, which, in addition to Dukes' stages A, B and C, defines tumours with distant organ metastases as stage D. Most of the patients (72%) were subjected to surgery alone. Radiotherapy or chemotherapy was used as adjuvant treatment for the remaining cases.

**Table I** Clinico-pathological characteristics of patients with colorectal cancer recruited by two national cancer institutes

| | *IRE*[a] *No. of cases* | *INT*[b] *No. of cases* |
|---|---|---|
| Case series | 71 | 110 |
| Sex | | |
| Male | 42 | 60 |
| Female | 29 | 50 |
| Age (median, years) | 61 | 60 |
| Site of primary | | |
| Right colon | 18 | 37 |
| Left colon | 26 | 47 |
| Rectum | 27 | 26 |
| Dukes' stage | | |
| A | 5 | 12 |
| B | 31 | 46 |
| C | 21 | 30 |
| D | 14 | 22 |
| Treatment | | |
| Surgery | 55 | 75 |
| Surgery + radiotherapy | 12 | 15 |
| Surgery + chemotherapy | 4 | 20 |

[a]Istituto Regina Elena, Rome; [b]Istituto Nazionale Tumori, Milan.

All patients, after surgical treatment, were followed as outpatients every 4 months for the first 2 years, every 6 months for the 3rd year, and yearly after the 5th year. Periodic controls consisted of a physical examination, colonoscopy (yearly), abdominal ultrasonography (quarterly), chest X-ray (semi-yearly) and blood test including serum CEA and CA 19.9. CT scan or NMR was performed in case of suspected local or distant recurrence.

Patients with Dukes' stages B or C rectal tumours received postoperative adjuvant radiotherapy (50 Gy). About 10% of patients entered a clinical protocol (trial EORTC 40871) and were randomised to receive surgical treatment alone or intraportal perfusional chemotherapy with 5-fluorouracil for the first 7 postoperative days.

Flow cytometric analysis of DNA content was performed starting with fresh (at the IRE) or frozen (at the INT)

Correspondence: R. Silvestrini, Oncologia Sperimentale C, Istituto Nazionale Tumori, via Venezian 1, 20133 Milano, Italy.
Received 29 July 1992; and in revised form 1 December 1992.

**Table II** Flow cytometric analysis: comparison between the two national cancer institutes

| | *IRE, Rome* | *INT, Milan* |
|---|---|---|
| Tissue sample | Fresh | Frozen |
| Suspension preparation | Mechanical disaggregation | Mechanical disaggregation |
| Type of suspension | Cells | Nuclei |
| Fixation | Cold acetone-methanol | – |
| Staining | Propidium iodide, RNase | Propidium iodide, RNase, Nonidet P40 |
| Flow cytometer | FACS 420 | FACScan |

tumour material, with minimal differences in the subsequent procedures between the two Institutes, as reported in Table II. From three to nine fragments taken from different areas of the lesion were pooled for DNA analysis. Cell suspensions were prepared immediately after tumour excision by mincing the tissue in phosphate-buffered saline (PBS), pH 7.6, followed by filtration through a nylon mesh (pore size, 160 μm) and fixation in a cold acetone-methanol (1:4, v/v) and PBS solution. Nuclei suspensions were obtained by mincing thawed tumour material in PBS, followed by filtration through a nylon mesh (pore size, 160 μm). The sample was centrifuged for 10 min at 1,500 *g*, and the pellet was resuspended in PBS at 4°C. The number of cells or nuclei was counted at the microscope, and the concentration was adjusted to approximately $10^6$ ml$^{-1}$. The suspensions were run in duplicate, and to one of them human peripheral lymphocytes were added as an internal standard before staining. Samples were stained for 20–30 min (at room temperature, IRE, and at 4°C, INT) in a solution containing propidium iodide (50 μg ml$^{-1}$) and RNase (75 Ku ml$^{-1}$, IRE, and 100 Ku ml$^{-1}$, INT). Nonidet P40 (0.05%) was added to nuclei suspensions. Immediately before flow cytometric analysis, the samples were passed through a 40-μm filter. A minimum of 30,000 events for each sample was analysed by a FACS 420 (Becton Dickinson, Mountain View, CA, USA) or a FACScan (Becton Dickinson, San Jose, CA, USA) flow cytometer in a single-parameter, 256-channel, integrated fluorescence histogram.

**Figure 1** Relapse-free and overall survival in diploid (broken line) and aneuploid (solid line) tumours: INT, Milan experience.

**Table III** DNA ploidy in primary colorectal cancer: experiences of two national cancer institutes

| | *IRE, Rome (n = 71)* | *INT, Milan (n = 110)* |
|---|---|---|
| Aneuploidy | 63% | 66% |
| DNA index, median (range) | 1.6 (1.1–2.3) | 1.6 (0.9–2.8) |
| Multiploidy | 17% | 12% |

**Table IV** DNA ploidy in relation to clinico-pathological characteristics of primary colorectal cancer

| | *Incidence (%) of aneuploidy* | |
|---|---|---|
| | *IRE, Rome* | *INT, Milan* |
| Site of primary | | |
| Right colon | 52 | 51 |
| Left colon | 67 | 70 |
| Rectum | 60 | 80 |
| Dukes' stage | | |
| A | 33 | 83 |
| B | 67 | 59 |
| C | 48 | 70 |
| D | 88 | 68 |
| Histology | | |
| Adenocarcinoma: | | |
| Well differentiated | 63 | 63 |
| Moderately/poorly differentiated | 67 | 69 |
| Mucinous | 40 | 56 |

The mean coefficient of variation of the diploid peak was 5% (range, 3.5–7.8%) in the IRE series and 3.5% (range, 2.7–6.5%) in the INT series. DNA ploidy was defined as DNA index (DI), i.e., as the ratio between the mean channel number of the $G_{0/1}$ peak of tumour cells and that of lymphocytes. Tumours with a DNA index different from 1 were considered to be aneuploid. When two cell populations were present, the highest DNA index was used to define tumour ploidy; when three or more cell populations were present, the DNA index of the largest aneuploid population was chosen as representative of the tumour.

Differences in DNA ploidy patterns among the various clinico-pathological subsets were analysed by the chi-square and the Wilcoxon rank-sum tests. The Bonferroni procedure was adopted to test multiple comparisons. Relapse-free survival (RFS) and overall survival (OS) were computed, starting from the date of surgery, by means of the Kaplan-Meier (1958) product-limit method. The log rank test was used to assess differences among subgroups.

The role of ploidy as a prognostic variable (univariate analysis) was evaluated by resorting to a Weibull regression model. In this model each regression coefficient (β) is recognisable as the log of the hazard ratio and it is constant in time. For ploidy the unadjusted hazard ratio and its 95% confidence interval were estimated according to a regression model containing only that variable, by using the putative best prognosis as reference category.

**Figure 2** Relapse-free and overall survival in diploid (broken line) and aneuploid (solid line) tumours: IRE, Rome experience.

### Results

Superimposable frequencies of aneuploid tumours (63% and 66% for the IRE and INT, respectively) and the same median DI value (1.6) were observed for the two case series, irrespective of whether fresh or frozen tumour material was used. Multiploid tumours were present in 17% and 12% of the IRE and INT series, respectively (Table III).

DNA ploidy was analysed in relation to site of the primary tumour, Dukes' stage and histological grade (Table IV). No significant relation was observed between DNA ploidy of the primary tumour and any of the considered variables in either of the case series. However, a higher frequency of aneuploid tumours was observed in Dukes' stage D than in Dukes' stage A tumours (88% *vs* 33%) in the IRE case series.

The clinical outcome of patients in the two case series was analysed as a function of DNA ploidy. RFS and OS curves for patients with diploid or aneuploid tumours were superimposable in the Milan experience (Figure 1). In the IRE experience, slightly better (not statistically significant) RFS and OS were observed during the first 3 years for patients with diploid than for those with aneuploid tumours, but the clinical outcome was similar at 4 years (Figure 2). Similar findings were also reflected by the hazard ratios and their confidence limits for INT and IRE series (Table V). No additive prognostic information was obtained from a breakdown analysis as a function of various DI values and different percentages of aneuploid cells (data not shown).

Conversely, we observed a significantly better RFS in both IRE (78% *vs* 53%) and INT (55% *vs* 27%) series for patients with tumours showing a normal DNA content or only one aneuploid subpopulation than for patients with tumours made up of more than one aneuploid subpopulation. Similarly, OS was significantly better in the former than in the latter group (69% *vs* 33% and 63% *vs* 35%, respectively) (Figure 3). Hazard ratios and their confidence intervals are also reported in Table V to provide a quantitative measure of the effect of multiploid DNA content on RFS and OS. In both the case series the probability of relapse and death was higher for patients with multiploid tumours than for patients with diploid or aneuploid tumours.

### Discussion

Colorectal adenocarcinoma is estimated to have a high incidence and mortality among all cancer diagnoses in both sexes. Attempts to reduce mortality have taken many forms, such as identification of patients at different risk for whom there is the necessity to consider the role and the modality of adjuvant treatments. Numerous studies have verified the clinical utility of stage at diagnosis as the single most important prognostic indicator (Wiggers *et al.*, 1988).

However, considering the results obtained for other human tumour types, it could be supposed that biological characteristics, such as DNA ploidy, can give additional information to integrate with clinical or pathological staging. For colorectal cancer, the prognostic relevance of ploidy has not been consistently demonstrated by different studies, and the disagreement can be ascribed to heterogeneity of FC procedures, interpretation of results, and the case series analysed.

In this joint study of two national cancer institutes in Italy, superimposable results were independently obtained. The frequencies of aneuploid tumours in the two case series are in accord with the highest values reported in the literature, thus indicating that no aneuploid cell populations were lost during methodological manipulations, and even near-diploid populations were detected. Comparability of results was possible owing to the similar distributions of tumours in the different

**Table V** Univariate analysis of 4-year follow-up

| | Relapse Hazard ratio (95% CL) | $P^a$ | Death Hazard ratio (95% CL) | $P^a$ |
|---|---|---|---|---|
| *Aneuploid vs diploid*[b] | | | | |
| INT, Milan | 1.07 (0.53–2.12) | ns[c] | 1.20 (0.56–2.56) | ns |
| IRE, Rome | 2.08 (0.66–2.49) | ns | 1.48 (0.69–3.16) | ns |
| *Multiploid vs diploid or aneuploid*[b] | | | | |
| INT, Milan | 3.12 (1.34–7.27) | 0.008 | 3.24 (1.30–8.04) | 0.01 |
| IRE, Rome | 5.47 (1.60–8.73) | 0.007 | 2.53 (0.97–6.62) | 0.055 |

[a]Wald statistics. [b]Reference category. [c]ns, not significant.

**Figure 3** Relapse-free **a**, **b**, and overall survival **c**, **d**, evaluated on series of primary colorectal cancers from two national cancer institutes (broken line, diploid or aneuploid; solid line, multiploid).

Dukes' stages, the similar patient treatment, and the careful follow-up through periodic clinical and instrumental examinations by both the institutes.

In our multicentric study, DNA ploidy, different DI values, and percentage of aneuploid cells were all unable to identify subgroups of patients with different risks of relapse or death, in agreement with the results reported by Kouri *et al.* (1990) and Rognum *et al.* (1987) at a similar 4-year follow-up. Conversely, our data are in disagreement with most reported results of several studies with a longer follow-up (Armitage *et al.*, 1990; Harlow *et al.*, 1991; Rognum *et al.*, 1991). Such results appear to indicate that DNA ploidy could be a late indicator of survival, and this hypothesis is supported by the appearance of a prognostic relevance of DNA ploidy at 5 years (Rognum *et al.*, 1991) in the same series of patients in which clinical relevance had not been detected at a shorter follow-up (Rognum *et al.*, 1987). The long time required for DNA ploidy to exhibit its impact on survival is probably due to the limitation of a prognostic relevance to Dukes' stages A, B and C. Conversely, our results showed, for the first time in colorectal cancer, that multiploidy represents an early and eventually unfavourable prognostic factor.

In any case, in view of the biological heterogeneity of colorectal cancer, further analyses on subgroups of patients defined according to the most important prognostic factors, such as Dukes' stage, site of the primary and type of treatment, could even more accurately identify very high-risk patient groups.

This work was supported in part by grants of the Special Project 'ACRO' of the Consiglio Nazionale delle Ricerche, Rome, and of the Associazione Italiana Ricerca sul Cancro, Italy.

### References

ARMITAGE, N.C., BALLANTYNE, K.C, EVANS, D.F., CLARKE, P., SHEFFIELD, J. & HARDCASTLE, J.D. (1990). The influence of tumour cell DNA content on survival in colorectal cancer: a detailed analysis. *Br. J. Cancer*, **62**, 852-856.

BAUER, K.D., LINCOLN, S.T., VERA-ROMAN, J.M., WALLEMARK, C.B., CHMIEL, J.S., MADURSKI, M.L., MURAD, T. & SCARPELLI, D.G. (1987). Prognostic implications of proliferative activity and DNA aneuploidy in colonic adenocarcinomas. *Lab. Invest.*, **57**, 329-335.

CUSICK, E.L., MILTON, J.I. & EWEN, S.W.B. (1990). The resolution of aneuploid DNA stem lines by flow cytometry: limitations imposed by the coefficient of variation and the percentage of aneuploid nuclei. *Anal. Cell. Pathol.*, **2**, 139-148.

GIARETTI, W,. DANOVA, M., GEIDO, S., MAZZINI, G., SCIALLERO, S., ASTE, H., SCIVETTI, P., RICCARDI, A., MARSANO, B., MERLO, F. & D'AMORE, E.S.G. (1991). Flow cytometric DNA index in the prognosis of colorectal cancer. *Cancer*, **67**, 1921-1927.

HARLOW, S.P., ERIKSEN, B.L., POGGENSEE, L., CHMIEL, J.S., SCARPELLI, D.G., MURAD, T. & BAUER, K.D. (1991). Prognostic implications of proliferative activity and DNA aneuploidy in Astler-Coller Dukes stage C colonic adenocarcinomas. *Cancer Res.*, **51**, 2403-2409.

HEDLEY, D.W. (1989). Flow cytometry using paraffin-embedded tissue: five years on. *Cytometry*, **10**, 229-241.

JASS, J.R., MUKAWA, K., GOH, H.S., LOVE, S.B. & CAPELLARO, D. (1989). Clinical importance of DNA content in rectal cancer measured by flow cytometry. *J. Clin. Pathol.*, **42**, 254-259.

JONES, D.J., MOORE, M. & SCHOFIELD, P.F. (1988). Prognostic significance of DNA ploidy in colorectal cancer: a prospective flow cytometric study. *Br. J. Surg.*, **75**, 28-33.

KALLIONIEMI, O.P. (1988). Comparison of fresh and paraffin-embedded tissue as starting material for DNA flow cytometry and evaluation of intratumor heterogeneity. *Cytometry*, **9**, 164-169.

KAPLAN, E.L. & MEIER, P. (1958). Nonparametric estimation from incomplete observations. *J. Am. Stat. Assoc.*, **53**, 457-481.

KOKAL, W., SHEIBANI, K., TERZ, J. & HARADA, J.R. (1986). Tumor DNA content in the prognosis of colorectal carcinoma. *J. Am. Med. Assoc.*, **255**, 3123-3127.

KOURI, M., PYRHOENEN, S., MECKLIN, J.P., JARVINEN, H., LAASONEN, A., FRANSSILA, K. & MORDLING, S. (1990). The prognostic value of DNA-ploidy in colorectal carcinoma: a prospective study. *Br. J. Cancer*, **62**, 976-981.

QUIRKE, P., DIXON, M.F., CLAYDEN, A.D., DURDEY, P., DYSON, J.E.D., WILLIAMS, N.S. & BIRD, C.C. (1987). Prognostic significance of DNA aneuploidy and cell proliferation in rectal adenocarcinomas. *J. Pathol.*, **151**, 285-291.

ROGNUM, T.O., THORUD, E. & LUND, E. (1987). Survival of large bowel carcinoma patients with different DNA ploidy. *Br. J. Cancer*, **56**, 633-636.

ROGNUM, T.O., LUND, E., MELING, G.I. & LANGMARK, F. (1991). Near diploid large bowel carcinomas have better five-year survival than aneuploid ones. *Cancer*, **68**, 1077-1081.

SCHUTTE, B., REYNDERS, M.J., WIGGERS, T., ARENDS, J.W., VOLOVICS, L., BOSMAN, F.T. & BLIJHAM, G.H. (1987). Retrospective analysis of the prognostic significance of DNA content and proliferative activity in large bowel carcinoma. *Cancer Res*, **47**, 5494-5496.

WIGGERS, T., ARENDS, J.W., SCHUTTE, B., VOLOVICS, L. & BOSMAN, F.T. (1988). A multivariate analysis of pathological prognostic indicators in large-bowel cancer. *Cancer*, **61**, 386-395.